# Synthesis, Crystal Structure, and Kinetics of the Thermal Decomposition of the Nickel(II) Complex of the Schiff Base 2-[(4-Methylphenylimino)methyl]-6-methoxyphenol

**DOI:** 10.3390/molecules14072582

**Published:** 2009-07-15

**Authors:** Yan-Fang Wang, Jian-Feng Liu, Hui-Duo Xian, Guo-Liang Zhao

**Affiliations:** 1 Zhejiang Key Laboratory for Reactive Chemistry on Solid Surfaces, Institute of Physical Chemistry, College of Chemistry and Life Science, Zhejiang Normal University, Jinhua, 321004, China; 2 Ningbo Tian Yi College of Occupation and Technology, Ningbo, Zhejiang 315100, China; E-mails: yfw901@163.com (Y-F.W.); liujianfeng23@163.com (J-F.L.); hdxian@163.com (H-D.X.)

**Keywords:** dinickel complex, crystal structure, Schiff base

## Abstract

A new dinuclear complex, [Ni_2_(H_2_O)L_4_]**·**5H_2_O, consisting of chelating bidentate and bridging tridentate coordinated 2-[(4-methylphenylimino)methyl]-6-methoxyphenol (HL) Schiff base ligands and water molecules has been synthesized using a traditional method. The structure of this complex was characterized by FTIR and UV/Vis spectroscopy and thermogravimetric analyses (TG-DTG) and further confirmed by single-crystal X-ray diffraction. Its crystal structure is of monoclinic system, space group P21/c with a = 13.2837(5) Å, b = 27.3886(10) Å, c = 17.5415(6) Å, α = 90 º, β = 108.429(2) º, γ = 90 º, V = 6054.7(4) Å^3^, Z = 4. The crystal structure reveals that there is a Ni·Ni core, with a separation of 3.183 Å. Its thermal decomposition kinetics were also studied.

## 1. Introduction

Polynuclear transition metal complexes have been receiving considerable attention for many years, due to their interesting characteristics in the fields of material science and biological systems [1,2]. o-Vanillin is considered a safe food flavoring and can be used as a pharmaceutical intermediate [3,4], and because of the wide applications o-vanillin derivatives and their complexes have drawn much attention [5,6,7,8]. Schiff base ligands which usually contain O, N donor atoms have played an important role in coordination chemistry since the late 19th century. Metal complexes with these ligands are becoming increasingly important as biochemical, analytical and antimicrobial reagents, in the design of molecular magnets, materials chemistry and so on [9,10,11,12,13,14]. The versatile properties of the ligand 2-[(4-methyl-phenylimino)methyl]-6-methoxyphenol (HL) in the formation of diverse transition metal complexes are well known. We have synthesized some complexes from HL, with discrete, dinuclear and polynuclear structures [15,16,17]. In continuation of the research in our group, we now describe a detailed investigation on the synthesis, single X-ray crystallographic and kinetics of the thermal decomposition of [Ni_2_(H_2_O)L_4_]·5H_2_O (**1**).

## 2. Results and Discussion

### 2.1. IR spectra

Important information obtained from the IR spectra of HL and complex **1** is summarized in Table 1. The broad absorption bands at about 3,444 cm^-1^, attributed to the *v*(O-H), indicate the presence of water in this compound. In addition, the band appearing at 615 cm^-1^ and resulting from *δ*(O-H) bends also supports this conclusion. Some sharp bands appearing at about 2,962 cm^-1^ and 1,380 cm^-1^ belong to the *v*(C-H) and *δ*(C-H) of methyl group and methoxyl groups. The characteristic absorption bands resulting from the skeletal vibrations of the aromatic rings from the Schiff base ligands are in the 1,400-1,600 cm^-1^ region (1,597 cm^-1^, 1,508 cm^-1^ and 1,470 cm^-1^). The typical *v*(C=N) band at 1,641 cm^-1^ in the spectrum of HL changes to 1,619 cm^-1^ after the imine N bonds to the metal ion. The shift of the C–O stretching vibration of the phenolic part of *o*-vanillin from 1,257 cm^-1^ to 1,238 cm^-1^ also supports the coordination of phenolic oxygen atoms. At last, the new band appearing at 509 cm^-1^ is attributed to M–O stretching vibration, which is absent in ligand spectra [18].

**Table 1:** Values of IR spectra for complex **1** and HL (cm^-1^).

Compound	v OH	v C=N	v C-O	v M-O
HL	3468 (w)	1614 (s)	1257 (s)	—
[Ni2(H_2_O)L4]·5H_2_O	3444 (m)	1619 (s)	1238 (s)	509 (w)

* Note: s: strong, m: middle, w: weak.

### 2.2. UV/Vis spectra

**Figure 1:**
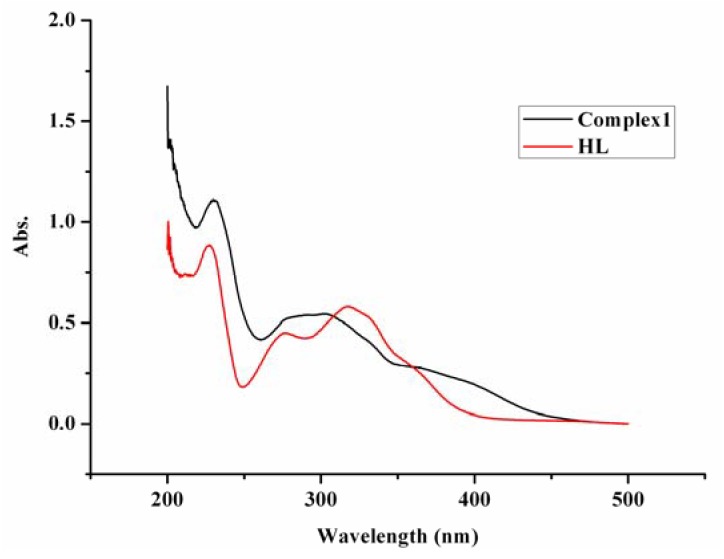
The UV/vis spectra of the complex **1 **and the ligand **HL**.

The absorptions in the UV/Vis spectra of HL and complex **1** are similar (Figure 1). There are three absorption peaks in the range of 200~500 nm at 317.8, 276.0 and 228.0 nm in the spectra of HL. The counterparts of the complex appear at 301.4, 292.0 and 229.8 nm. The first peak is assigned to n-π* transition of conjugation between lone-pair electron N atom in the C=N group and big π bond of benzene ring. The hypsochromic shift of about 16 nm is caused by the coordination of N atom to Ni ion, which can also provide the conclusion evidence for the coordination. The other two peaks are attributed to π-π* transition of the conjugation system of Schiff base and they are similar in HL and in the complex.

### 2.3. Crystal structure

The complex is an asymmetric dinuclear nickel complex and there is no significant metal-metal interaction between the two nickel centers considering of the separation of Ni(1)···Ni(2) is 3.1831(3) Å, which is longer than that of previously reported dinickel complexes [19]. The environments of the two nickel atoms are very similar to each other; both are six-coordinate, coordinated to four oxygen and two nitrogen atoms in a slightly distorted octahedron, although there are still some differences such as Ni(1) is bound by four O atoms (O4, O7, O10, O1w) coming from three different phenolic groups of Schiff base (L1, L2, L3) ligands and a coordinated water molecule, where the Ni–O bond distances range from 1.9410(10) to 2.3389(10) Å, whereas the four O atoms that surround Ni(2) come from three Schiff base molecules (L1, L3, L4), and the bond distances are from 2.0048(10) to 2.2876(9) Å. To the best of our knowledge, this is the first example of a crystallographically characterized complex with four 2-[(4-methylphenylimino)methyl]-6-methoxyphenol Schiff base ligands with three types of coordination modes in one complex: (i) simple N,O-chelating bidentate [Figure 3 (a)] L2 and L4 belong to this mode, and they chelate to Ni(1) and Ni(2) respectively, the coordinated atoms are both phenolic O and imino N; (ii) bidentate [Figure 3 (b)] L3 chelates to Ni(2) with phenolic O and imino N, and the phenolic O also bridges to Ni(1); (iii) bridging quadridentate [Figure 3 (c)], this mode is adopted by L1. In addition to the phenolic O and imino N, the methoxyl O is also involved in the coordination to Ni. These are apparently important for rational design and constitution of new framework structures. The main bond lengths and bond angles are listed in Table 7. Two methoxy-phenol rings of the L2 and L3 ligand are almost parallel to each other with a dihedral angels 13.91°, and the center distance of which is 3.85 Å indicated a weak π···π stacking interaction [20]. There are several water molecules involved in O–H···O intramolecular hydrogen-bonding interactions, which help to stabilize this structure. 

**Figure 2:**
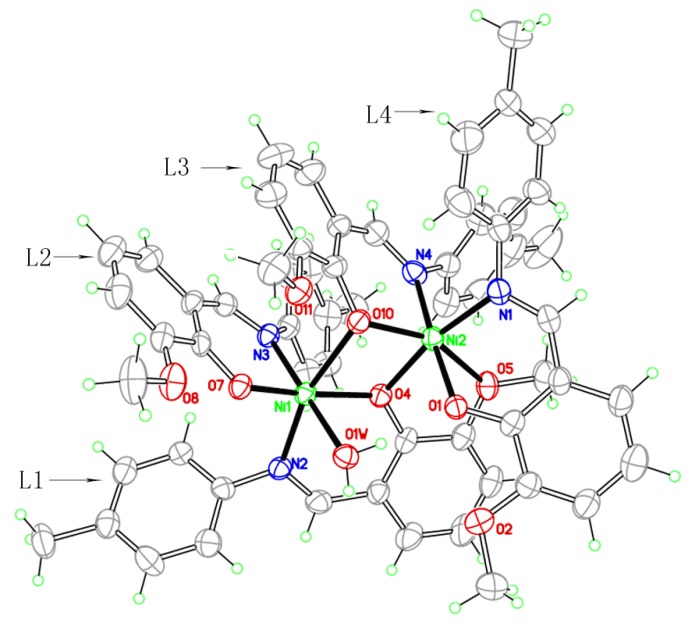
Molecular structure of the complex **1** (probability of ellipsoid is 30%). All the non-coordinated water molecules are omitted for clarity.

**Figure 3:**
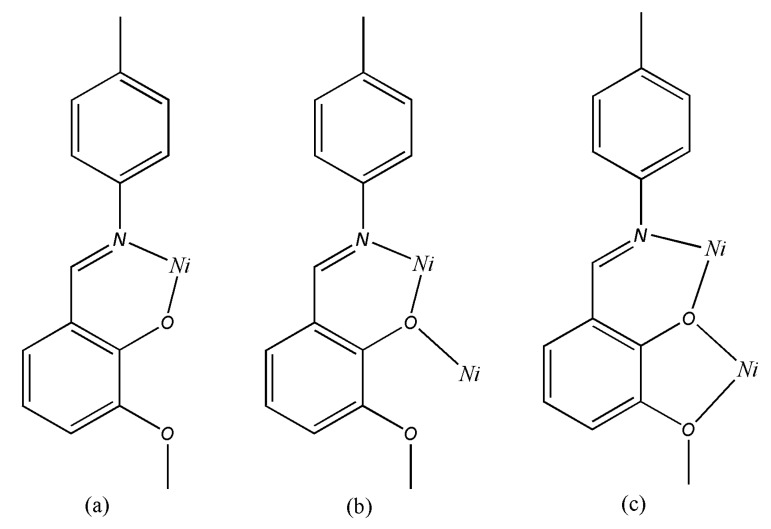
The three coordinate modes of L in complex **1.**

### 2.4. Thermal analysis

**Figure 4:**
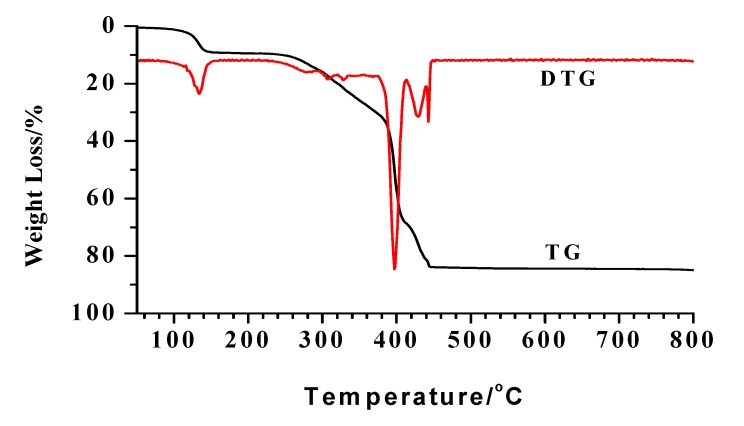
The TG-DTG curves of complex **1.**

The TG-DTG curve of complex **1** is shown in Figure 4. There are three main weight loss stages for the decomposition process. In the first stage, from 76 ^◦^C~152 ^◦^C, 9.14% of the weight is lost, which corresponds to the loss of six water molecules, which coincides with the calculated value 9.10%. The temperature range of 226 °C~413 °C is the second stage, with a weight loss of about 59.46% and it seems that three Schiff ligand molecules has burned off (calcd. 61.00%). The remaining one cannot remain too long, and its loss accounts for the third stage with a loss of 15.60% in the temperature range of 431 ^◦^C ~460 ^◦^C (the calculated value is 13.95%). The remaining mass of 15.50% seems likely to correspond to NiO (calcd. 12.56%).

### 2.5. Non-isothermal kinetics for Steps 1 and 2

For the TG and DTG curves, the non-isothermal kinetic data in the steps 1 and 3 of the thermal decomposition were analyzed by means of differential and integral methods. The differential equation used was proposed by Achar et al [21,22]. The integral equation was Coats-Redfern equation [23]. The used forms f(α) and g(α) are listed in Table 2. The original data for Steps 1 and 3 determined from the TG-DTG curves are listed in Table 3.

Archar equation:

ln[(dα/dt)/f(α)] =lnA - E/(RT)

Coats-Redfern equation:

ln[g(α)/T^2^] = ln(AR)/(βE) - E/(RT)

In the above equations, α is the fraction of the reacted material, T is the absolute temperature and f(α) and g(α) are differential and integral mechanism functions, respectively. E and A are the derived apparent activation energy and pre-exponential factor, respectively, R is the gas constant and β is the heating rate.

The kinetic analysis was performed by the linear least square method. The results of the kinetic parameters E, A and correlation coefficients r for step 1 and 2 are shown in Table 3.

We fitted the kinetic parameters obtained (E and A) using the equations of Achar *et al*. and Coats-Redfern (see Table 4). Comparing the results from the differential and integral methods, we found that the values of E and A were acceptable and the linear correlation coefficients were also better when the possible mechanism functions were rational selected.

**Table 2:** Some integral and differential coefficient kinetic functions.

Symbol	Integral kinetic function	Differential coefficient kinetic function	Mechanism
D1	α^2^	1/(2α)	One-dimensional diffusion
D2	α+(1-α)ln(1-α)	[-ln(1-α)]^-1^	Two-dimensional diffusion
1D3	1-(2/3)α-(1-α)^2/3^	3/2[(1-α)^-1/3^-1]^-1^	Three-dimensional diffusion (cylindrical symmetry)
2D3	[1-(1-α)^1/3^]^2^	3/2(1-α)^2/3^[1-(1-α)^1/3^]^-1^	Three-dimensional diffusion (spherical symmetry)
3D3	[(1+α)^1/3^-1]^2^	3/2(1+α)^2/3^[(1+α)^1/3^-1]^-1^	Three-dimensional diffusion
4D3	[1/(1-α)^1/3^-1]^1/2^	3/2(1-α)^4/3^[1/(1-α)^1/3^-1]^-1^	Three-dimensional diffusion
A1	-ln(1-α)	(1-α)	Random nucleation and nuclei growth (n=1)
A1.5	[-ln(1-α)]^2/3^	3/2(1-α)[-ln(1-α)]^1/3^	Random nucleation and nuclei growth (n=1.5)
A2	[-ln(1-α)]^1/2^	2(1-α)[-ln(1-α)]^1/2^	Random nucleation and nuclei growth (n=2)
A3	[-ln(1-α)]^1/3^	3(1-α) [-ln(1-α)]^2/3^	Random nucleation and nuclei growth (n=3)
A4	[-ln(1-α)]^1/4^	4(1-α) [-ln(1-α)]^3/4^	Random nucleation and nuclei growth (n=4)
R2	1-(1-α)^1/2^	2(1-α)^1/2^	Contracting sphere (cylindrical symmetry)
R3	1-(1-α)^1/3^	3(1-α)^2/3^	Contractingsphere (spherical symmetry)
P1	α	1	Exponential nucleation
P2	α^1/2^	2α^1/2^	Exponential nucleation
P3	α^1/3^	3α^2/3^	Exponential nucleation
P4	α^1/4^	4α^3/4^	Exponential nucleation
C2	(1-α)^-1^-1	(1-α)^2^	Chemical reaction
C1.5	(1-α)^-1/2^	2(1-α)^3/2^	Chemical reaction

**Table 3:** Kinetic compensation parameters for Step 1 and Step 2.

Step 1			Step 2		
T/K	α	dα/dt	T/K	α	dα/dt
363.15	0.0204	2.1941	493.15	0.0158	2.5213
373.15	0.0548	1.8824	503.15	0.0416	1.3356
383.15	0.1163	1.5496	513.15	0.0930	1.8930
393.15	0.2237	1.6131	523.15	0.1888	1.6043
403.15	0.4937	1.8258	533.15	0.3547	1.3785
413.15	0.9043	0.6285	543.15	0.6250	1.2225

**Table 4:** The kinetic data of thermal decomposition of Step 1.

	Achar method			Coats–Redfern method		
No.	*E*/kJ mol^-1^	A/s^-1^	*r*	*E*/kJ mol^-1^	*A*/s^-1^	*r*
1	207.59	2.18E+06	0.9548	171.38	3.37E+20	0.9989
2	265.51	4.98E-03	0.9914	181.63	5.40E+21	0.9990
3	290.10	8.78E+02	0.9947	185.89	5.06E+21	0.9986
4	336.58	8.53E-12	0.9905	194.64	9.66E+22	0.9970
5	162.82	4.90E-14	0.9032	161.29	1.20E+18	0.9980
6	475.99	2.52E+00	0.9573	224.61	2.31E+27	0.9867
7	74.73	1.27E+02	0.7679	101.24	3.26E+11	0.9926
8	-35.34	1.16E-05	0.5499	65.41	3.97E+06	0.9922
9	36.58	2.58E-16	0.6520	47.49	1.25E+04	0.9918
10	-145.40	6.08E-20	0.9530	29.57	3.38E+01	0.9907
11	-172.92	8.57E-22	0.9683	20.61	1.55E+00	0.9894
12	5.02	4.07E+17	0.1679	90.98	4.87E+09	0.9981
13	28.25	4.70E+11	0.6219	94.19	9.74E+09	0.9969
14	-64.69	1.16E-05	0.7463	82.55	5.37E+08	0.9988
15	-200.83	2.58E-16	0.9640	38.14	4.51E+02	0.9986
16	-246.21	6.08E-20	0.9761	23.34	3.37E+00	0.9983
17	-268.90	8.57E-22	0.9799	15.94	2.54E-01	0.9979
18	214.14	4.07E+17	0.7969	127.51	2.47E+15	0.9673
19	144.43	4.70E+11	0.7922	16.21	6.03E-01	0.6840

Thus we deduced that the possible mechanism for the step 1 of thermal decomposition of the complex was a 1D3 chemical reaction. The corresponding non-isothermal kinetic equation was:

f(α)=2/3[(1-α)^-1/3^-1]^-1^, g(α)=1-(2/3)α-(1-α)^2/3^

The average values of the apparent activation energy E and pre-exponential factor A were 239.00 kJ·mol^-1^ and 0.9967, respectively.

The possible mechanism for step 2 was 2D3 chemical reaction. The corresponding non-isothermal kinetic equation was

f(α)=3/2(1-α)^2/3^[1-(1-α)1/3]-1, g(α)=[1-(1-α)1/3]^2^

The average values of the apparent activation energy E and pre-exponential factor A were 376.34 kJ·mol^-1^ and 0.9962, respectively.

From the thermal analysis, we can see the decomposition procession of the complex is complicated. For infer the mechanism of the thermal decomposition, we analyzed the non-isothermal kinetic in two decomposition steps by means of differential and integral methods. We have found the proper mechanism functions of the two steps and their decomposition activation energy.

**Table 5:** The kinetic data of thermal decomposition of Step 2.

	Achar method			Coats–Redfern method		
No.	E/kJ mol^-1^	A/s^-1^	r	E/kJ mol^-1^	A/s^-1^	r
1	325.14	1.12E+21	0.9875	317.17	3.75E+28	0.9974
2	366.94	3.70E+23	0.9927	328.16	2.72E+29	0.9985
3	382.33	2.02E+24	0.9933	332.19	1.61E+29	0.9988
4	412.37	9.59E+25	0.9932	340.31	1.16E+30	0.9992
5	276.76	6.57E+16	0.9760	302.95	1.28E+26	0.9958
6	502.52	1.16E+32	0.9872	365.92	5.83E+32	0.9994
7	35.18	4.55E+02	0.4475	172.10	4.57E+14	0.9995
8	-105.45	3.05E-07	0.8205	111.86	4.83E+08	0.9995
9	2.73	1.46E+00	0.0447	81.75	4.50E+05	0.9994
10	-246.09	1.53E-16	0.9545	51.63	3.63E+02	0.9994
11	-281.25	6.48E-19	0.9638	36.57	9.18E+00	0.9993
12	-9.90	2.07E-01	0.1732	162.85	2.34E+13	0.9988
13	5.13	1.42E+00	0.0849	165.85	3.27E+13	0.9992
14	-54.97	3.76E-04	0.7347	154.28	5.67E+12	0.9972
15	-245.03	1.54E-16	0.9743	72.84	4.72E+04	0.9968
16	-308.38	9.63E-21	0.9820	45.69	7.71E+01	0.9963
17	-340.06	6.99E-23	0.9844	32.12	2.77E+00	0.9958
18	125.33	5.51E+08	0.7525	192.70	7.18E+16	0.9979
19	80.25	2.50E+05	0.6703	10.60	1.22E-02	0.7115

## 3. Experimental

### 3.1. Materials and general methods

NiCl_2_·6H_2_O, *o*-vanillin, *p*-toluidine, and other chemical reagents were obtained from commercial sources and used without further purification. The metal contents were determined by EDTA complexometric titration after decomposition a known amount of the complexes with concentrated nitric acid. Elemental analyses for C, H and N were carried out on an Elementar Vario EL III elemental analyzer. IR spectra of KBr pellets were recorded on a Nicolet NEXUS 670 FTIR spectrophotometer in the range of 4,000-400 cm^-1^. Thermal analyses were carried out using Mettler-Toludo TGA/SDTA851^e^ thermal analyzer with a heating rate of 10 ^◦^C·min^-1^ from 30 ^◦^C to 800 ^◦^C in an air atmosphere. 

### 3.2. Syntheses

C_15_H_15_NO_2_(HL): Schiff base ligand was prepared by the direct solution reaction of *p*-toluidine (10.0 mmol, 1.07 g) and *o*-vanillin (0.01 mol, 1.52 g) refluxed in ethanol (~60 mL) on a water bath for 2 h. The solution was cooled to room temperature, and then the red crystals were obtained, washed with absolute ethanol and dried. Yield 1.93 g (80%), m.p.~100°C. It was recrystallized from methanol before used. 

**Figure 5:**
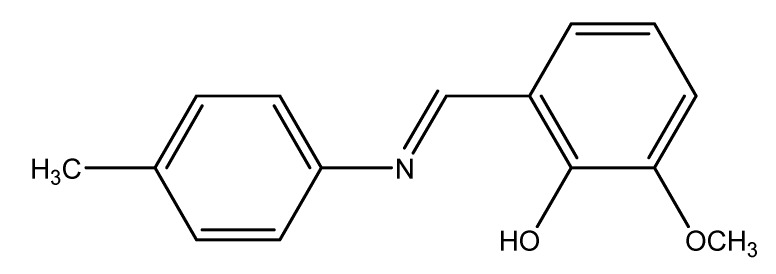
Structure of Schiff base ligand.

[Ni_2_(H_2_O)L_4_]**·**5H_2_O(**1**): NiCl_2_**·**6H_2_O (1 mmol, 0.237 g) dissolved in anhydrous ethanol (10 mL) was stirred with HL (0.57 g, 2 mmol) in anhydrous ethanol (20 mL) and refluxed for 2 h on a water bath, then the solid that deposited after cooling to room temperature was washed with ethanol and dried. Yield 0.80 g (70%). Green single crystals of complex **1** were obtained suitable for X-ray diffraction from the mother liquor after five days of slow evaporation at room temperature.

### 3.3. Crystal structure determination

Single crystals of complex **1** was selected and was structurely mesured measured with a Rigaku R-AXIS RAPID diffractometer with graphite-monochromated Mo-K*α* radiation (λ = 0.71073 Å ) at 296 K. Empirical absorption corrections were applied by use of the *ABSCOR* program. The structures were solved by direct methods and all calculations were performed with the aid of the SHELXL PC program [24]. The structures were refined by full-matrix, least-squares minimization of Σ(F_o_—F_c_)^2^ with anisotropic thermal parameters for all atoms except H atoms. The crystal data of the complex **1 **is summarized in Table 6, selected bond lengths and angles are show in Table 7.

**Table 6:** Crystallographic Data for Complex **1.**

Empirical formula	C_60_H_6__8_N_4_Ni_2_O_14_
Formula weight	1186.60
T/ K	296(2)
Crystal system	monoclinic
Space group	P 21/c
*a* (Å)	13.2837(5)
*b* (Å)	27.3886(10)
*c* (Å)	17.5415(6)
*α* (°)	90
*β* (°)	108.429(2)
*γ* (°)	90
*V* (Å^3^)	6054.7(4)
*Z*	4
Density (g/cm^3^)	1.302
*Μ* (mm^-1^)	0.687
*F* (000)	2496
Absorption correction	none
Data/restrains/parameters	10668 / 18 / 748
*θ* range /°	1.43 to 25.00
Limiting indices	-15<=h<=15, -32<=k<=31, -20<=l<=20
Reflections collected/ unique	81485 / 10668
R_int_	0.0950
*GOOF* on *F*^2^	1.032
*R* and *wR* (*I > 2σ(I)*)	*R* = 0.0873, *wR* = 0.2594
*R* indices (all data)	*R* = 0.1287, *wR* = 0.3012
(Δρ)_max_, (Δρ)_min_ (e·Å^-3^)	2.174 and -0.465

**Table 7:** Selected Bond Lengths (Å) and Bond Angles (°) for Complex **1.**

Bond	(Å)	Bond	(Å)	Bond	(Å)
Ni(1)-O(4)	1.9734(10)	C(7)-N(1)	1.2942(19)	C(18)-O(5)	1.3658(19)
Ni(1)-O(7)	1.9410(10)	C(9)-N(1)	1.4138(19)	C(23)-O(5)	1.450(2)
Ni(1)-O(10)	2.3389(10)	C(22)-N(2)	1.310(2)	C(32)-O(7)	1.3041(16)
Ni(1)-O(1W)	2.2364(8)	C(24)-N(2)	1.410(2)	C(33)-O(8)	1.3614(15)
Ni(1)-N(2)	2.0680(11)	C(39)-N(3)	1.4309(18)	C(38)-O(8)	1.436(2)
Ni(1)-N(3)	2.0898(10)	C(37)-N(3)	1.287(2)	C(47)-O(10)	1.3431(16)
Ni(2)-O(1)	2.0253(8)	C(52)-N(4)	1.321(2)	C(48)-O(11)	1.3759(15)
Ni(2)-O(4)	2.0602(10)	C(54)-N(4)	1.428(2)	C(53)-O(11)	1.4333(19)
Ni(2)-O(5)	2.2876(9)	C(2)-O(1)	1.3079(18)		
Ni(2)-O(10)	2.0048(10)	C(3)-O(2)	1.351(2)		
Ni(2)-N(1)	2.0977(13)	C(8)-O(2)	1.451(2)		
Ni(2)-N(4)	2.0545(10)	C(17)-O(4)	1.3049(16)		
Angle	(°)	Angle	(°)	Angle	(°)
O(4)-Ni(1)-O(10)	77.63(4)	N(3)-Ni(1)-O(4)	96.47(4)	O(10)-Ni(2)-O(4)	83.91(4)
O(4)-Ni(1)-O(1W)	86.08(4)	N(3)-Ni(1)-O(7)	91.81(4)	N(1)-Ni(2)-O(1)	83.81(4)
O(7)-Ni(1)-O(4)	165.05(4)	N(3)-Ni(1)-O(10)	94.16(4)	N(1)-Ni(2)-O(4)	169.18(4)
O(7)-Ni(1)-O(10)	89.40(4)	N(3)-Ni(1)-O(1W)	176.05(4)	N(1)-Ni(2)-O(10)	103.13(4)
O(7)-Ni(1)-O(1W)	85.06(4)	N(2)-Ni(1)-N(3)	98.66(4)	N(1)-Ni(2)-O(5)	100.19(4)
O(10)-Ni(1)-O(1W)	83.41(3)	O(1)-Ni(2)-O(4)	87.80(4)	N(4)-Ni(2)-O(1)	176.94(5)
N(2)-Ni(1)-O(4)	92.08(4)	O(1)-Ni(2)-O(5)	84.21(3)	N(4)-Ni(2)-O(4)	93.60(4)
N(2)-Ni(1)-O(7)	98.97(4)	O(4)-Ni(2)-O(5)	72.08(4)	N(4)-Ni(2)-O(5)	93.62(4)
N(2)-Ni(1)-O(10)	164.41(4)	O(5)-Ni(2)-O(10)	155.78(4)	N(4)-Ni(2)-O(10)	91.00(4)
N(2)-Ni(1)-O(1W)	84.23(4)	O(10)-Ni(2)-O(1)	91.85(4)	N(4)-Ni(2)-N(1)	94.47(5)

## 4. Supplementary Material

Supplementary crystallographic data have been deposited with the Cambridge Crystallographic Data Centre, CCDC No. 731857. Copies of this information may be obtained free of charge from The Director, CCDC, 12 Union Road, Cambridge CB2 1EZ, UK (Fax: +44 1223 336033; Email: deposit@ccdc.cam.ac.uk or www: http://www.ccdc.cam.ac.uk).
